# Involvement of the bed nucleus of the stria terminalis in L-Dopa induced dyskinesia

**DOI:** 10.1038/s41598-017-02572-9

**Published:** 2017-05-24

**Authors:** Matthieu F. Bastide, Christelle Glangetas, Evelyne Doudnikoff, Qin Li, Mathieu Bourdenx, Pierre-Olivier Fernagut, Éric C. Dumont, François Georges, Erwan Bézard

**Affiliations:** 1 Univ. de Bordeaux, Institut des Maladies Neurodégénératives, UMR 5293, F-33000 Bordeaux, France; 2 CNRS, Institut des Maladies Neurodégénératives, UMR 5293, F-33000 Bordeaux, France; 3 Motac neuroscience Ltd, Manchester, UK; 4 Institute of Lab Animal Sciences, China Academy of Medical Sciences, Beijing, China; 5 Department of Biomedical and Molecular Sciences, Queen’s University, Kingston, Canada

## Abstract

A whole brain immediate early gene mapping highlighted the dorsolateral bed nucleus of the stria terminalis (dlBST) as a structure putatively involved in L-3,4-dihydroxyphenylalanine (L-Dopa)-induced dyskinesia (LID), the debilitating side-effects of chronic dopamine replacement therapy in Parkinson’s disease (PD). dlBST indeed displayed an overexpression of ∆FosB, ARC, Zif268 and FRA2 only in dyskinetic rats. We thus hypothesized that dlBST could play a role in LID hyperkinetic manifestations. To assess the causal role of the dlBST in LID, we used Daun02 inactivation to selectively inhibit the electrical activity of dlBST ΔFosB-expressing neurons. Daun02 is a prodrug converted into Daunorubicin by ß-galactosidase. Then, the newly synthesized Daunorubicin is an inhibitor of neuronal excitability. Therefore, following induction of abnormal involuntary movements (AIMs), 6-OHDA rats were injected with Daun02 in the dlBST previously expressing ß-galactosidase under control of the FosB/ΔFosB promoter. Three days after Daun02 administration, the rats were tested daily with L-Dopa to assess LID. Pharmacogenetic inactivation of ∆FosB-expressing neuron electrophysiological activity significantly reduced AIM severity. The present study highlights the role of dlBST in the rodent analog of LID, offering a new target to investigate LID pathophysiology.

## Introduction

**Figure 1:**
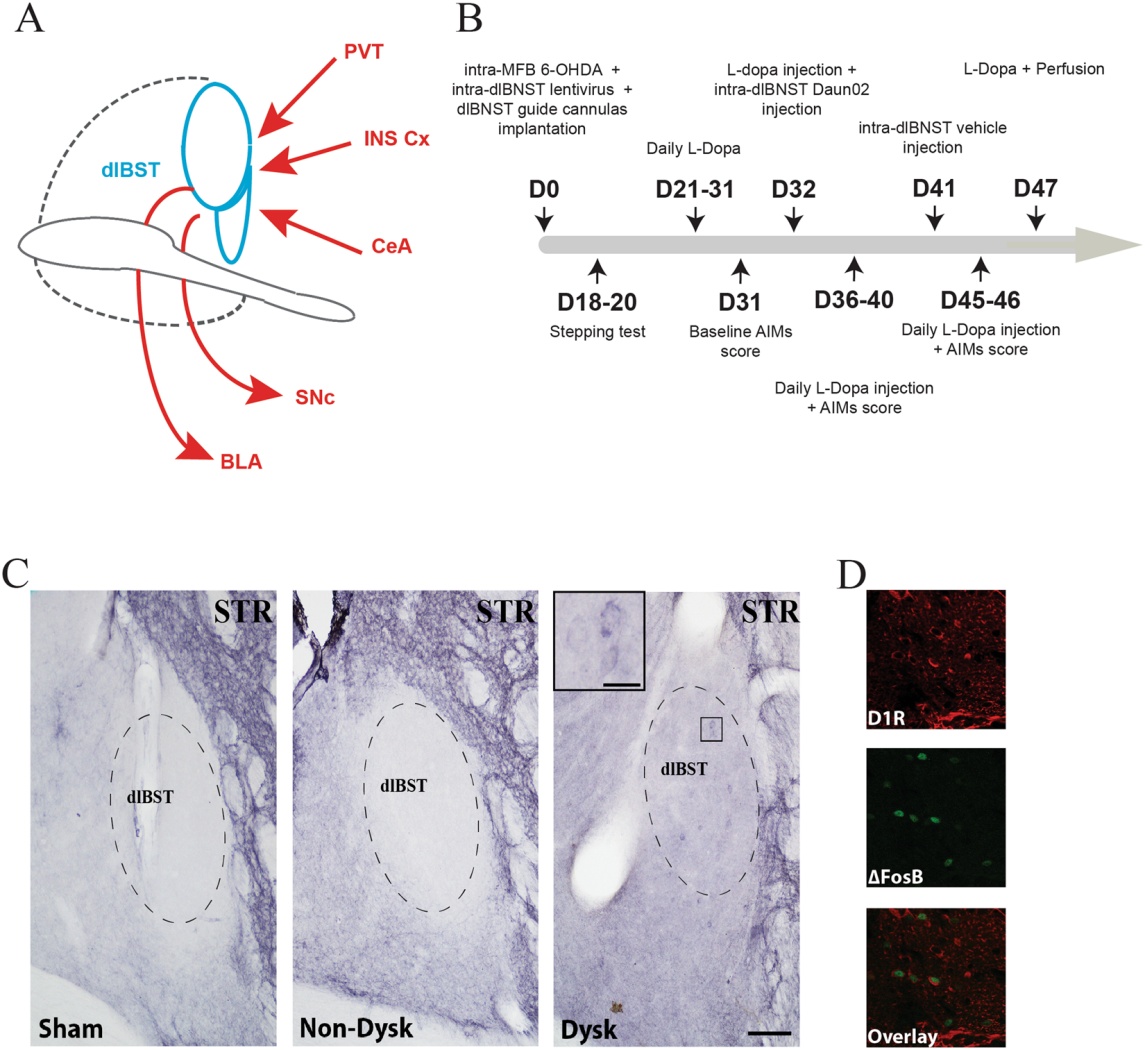
D1R expression in the dlBST. (**A**) Schematic summary of major known connections of the dlBST. BLA, basolateral nucleus of the amygdala; CeA, central nucleus of the amygdala; INS Cx, insular cortex; PVT, paraventricular nucleus of the thalamus; SNc, substantia nigra *pars compacta* ^24, 43, 44^. (**B**) Timeline of experimental manipulations. D, day. (**C**) Representative dlBST mapping of D1R expression (dashed lines) in sham-operated (sham), 6-OHDA-lesioned (Non-Dysk) and L-Dopa-treated dyskinetic 6-OHDA-lesioned rats (Dysk) (scale bar: 300 µm) with an inset showing a magnification of D1R expression in dyskinetic condition (scale bar: 5 µm) (STR = Striatum). (**D**) Representative insets (scale bar: 20 µm) showing D1R, ∆FosB and co-localization of D1R/∆FosB expression.

The gold standard treatment for Parkinson’s disease (PD) remains the dopamine precursor L-3,4-dihydroxyphenylalanine (L-Dopa). Long-term L-Dopa treatment systematically leads to abnormal involuntary movements (AIMs) called L-Dopa-induced dyskinesia (LID)^1^. From the 90’s to nowadays, growing evidence suggest that the mechanisms underlying PD and LID pathophysiology involve not only motor regions but also associative and limbic domains of the basal ganglia and beyond^1–4^, notably the bed nucleus of the stria terminalis (BST)^2^ (Fig. 1A).

Recently, a whole brain search approach highlighted the dorsolateral (dl) BST, which displayed an overexpression of 4 independent immediate early genes (IEG): ∆FosB, ARC, Zif268 and FRA2^5^ only in dyskinetic 6-OHDA-lesioned rats. The dlBST is composed of 2 nuclei, the oval (ovBST) and juxta (jxBST), both of which showed a significant correlation between ∆FosB or FRA2 expression and LID severity^5^. Based on this evidence, we hypothesize that the dlBST could be actively involved in LID manifestations.

Interestingly, striatal down-regulation of FosB expression or electrical inhibition of FosB/∆FosB-expressing neurons decreased LID severity both in rats and non-human primates^6–8^, demonstrating that FosB/∆FosB is not only a marker of LID but that inhibiting its expression or the electrical activity of the neurons expressing it functionally impact AIMs or LID. Moreover, similar results were recently obtained following inactivation of FosB/∆FosB-expressing neurons in a structure located outside of the basal ganglia (e.g. the lateral habenula) highlighting the involvement of extra-basal ganglia nuclei in LID manifestations^9^.

Therefore, to assess the role of the dlBST in LID pathophysiology, we used the FosB promoter to selectively drive the expression of ß-galactosidase in FosB/ΔFosB-expressing neurons. Then, we assessed the role of these ΔFosB-expressing dlBST neurons in the rat model of LID in PD^8–11^ by inhibiting their electrical activity with the Daun02-inactivation method^8, 9, 12–15^.

## Results

### LID induce D1R expression in the dlBST

Consistent with previous observations in the basal ganglia, we also found an increased expression of the dopaminergic D1 receptor (D1R) protein in the dlBST of dyskinetic rats (Fig. 1C), which co-localized with ∆FosB (Fig. 1D).

### Pharmacogenetic inhibition of dlBST ∆FosB-expressing neurons activity alleviated AIMs

**Figure 2:**
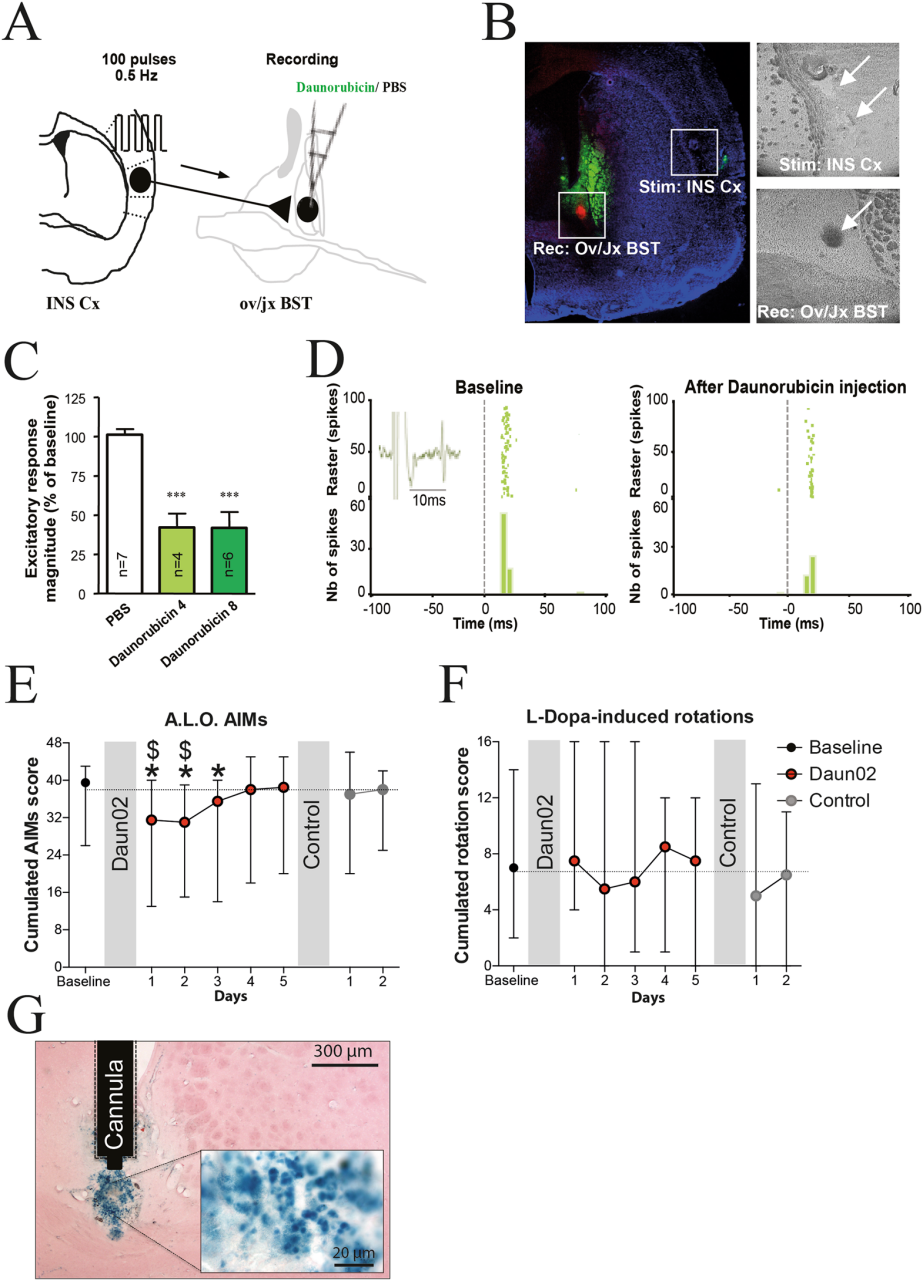
Daun02-induced inactivation of ∆FosB-expressing dlBST neurons alleviates LID in rats. (**A**) INS Ctx stimulation and ov/jxBST recording protocols. (**B**) Histological controls of stimulation site (stim: INS Ctx), recording site (rec: ov/jx BST; red fluorescent spot), injection site (green fluorescent labeling) (scale bar 1 mm) with 2 representative insets indicating the respective sites with white arrows (scale bar 0.6 mm). (**C**) Quantitative analysis of inhibitions induced by Daunorubicin infusion (Daunorubicin 4: 4 µg/µL; Daunorubicin 8: 8 µg/µL) on excitatory responses evoked by the INS Ctx stimulation. Only neurons responding to Daunorubicin have been included in this analysis (4 out of 7 for Daunorubicin 4 and 6 out of 6 for Daunorubicin 8) (***p < 0.001 from PBS). (**D**) Typical PSTHs and associated rasters showing responses of ov/jx BST neurons before and after daunorubicin (4 µg/µL) infusion. Stimulus at t_0_ (gray line). Bin width, 5 ms. Representative electrophysiological trace in inset. (**E**) Cumulated axial, limb and orolingual (A.L.O.) AIMs scores in L-Dopa-treated 6-OHDA rats (n = 10) before and after Daun02 and after control solution injection (median ± range; *p < 0.05 from baseline and ^$^p < 0.05 from control solution). (**F**) Cumulated rotation scores in L-Dopa-treated 6-OHDA rats (n = 10) before and after Daun02 and after control solution injection (median ± range). (**G**) Representative dlBST cytochemical detection of ß-galactosidase expression in the Daun02-injected side of dyskinetic rats (scale bar: 300 µm) with an inset (scale bar: 20 µm).

To directly assess a causal role of dlBST in AIM severity in the rodent analogue of dyskinesia, we inhibited the electrical activity of dlBST ∆FosB-expressing neurons using the selective Daun02/ß-galactosidase inactivation method. The prodrug Daun02 was locally administrated and presumably converted into Daunorubicin by ß-galactosidase, readily produced in neurons expressing the *E. coli* LacZ gene under the FosB/∆FosB gene promoter^8, 9, 12–14^. Then, the newly synthesized Daunorubicin is a potent inhibitor of neuronal excitability by reducing calcium ion-dependent action potentials^15^. We recently showed that Daun02/ß-galactosidase inactivation (genetic manipulation) or Daunorubicin injection (pharmacological manipulation) inhibit striatal neurons’ activity both *in vitro* and *ex vivo* ^8^. We further demonstrated that this inhibition is reversible following Daun02 washout^8^. Using *in vivo* single-unit extracellular recordings, we demonstrate that intra-dlBST injection of Daunorubicin (Fig. 2A,B) induced a 60% decrease in the number of evoked spikes in response to insular Ctx stimulation (Fig. 2C,D, Percentage of change between before and after values in Rmag: PBS = 101.3 ± 3.36%, n = 7 cells; Daunorubicin 4 µM = 42.20 ± 8.8%, n = 4 cells; Daunorubicin 8 µM = 41.85 ± 10.03%, n = 6 cells. One way Anova followed by a bonferonni post hoc: F_(2.14)_ = 22.88, p < 0.0001). No change was observed on basal spontaneous activity after intra-dlBST injection of PBS or Daunorubicin (PBS: before = 0.42 Hz ± 0.27 Hz, after = 0.17 Hz ± 0.12 Hz, N = 6 neurons, paired t test, p = 0.1663; Daunorubicin 4 µM = before: 2.08 Hz ± 1.99 Hz, after = 2.29 Hz ± 2.2 Hz, N = 4 neurons, paired t test; p = 0.4; Daunorubicin 8 µM: before = 0.34 Hz ± 0.15 Hz, after: 0.53 Hz ± 0.39 Hz, N = 4 neurons, paired t test; p = 0.53). All together, these results demonstrate that intra-dlBST injection of Daunorubicin target the synaptic activity by decreasing the excitability of dlBST neurons (decrease in the number of evoked spikes during each train of stimulation of the INS Ctx) without affecting their basal activity.

Therefore, we injected, *in vivo*, a FosB-LacZ lentivirus expressing the ß-galactosidase only in the dlBST^8, 9^ of 6-OHDA-lesioned rats chronically treated with L-Dopa^5, 10, 11, 16^. Due to the small size of the dlBST and the adjoining internal capsula, all viral infusions were performed under *in vivo* electrophysiological guidance using small volume of virus solution (250 nl) to limit virus particles diffusion to adjacent fiber bundles. After the establishment of stable AIMs, a single intra-dlBST administration of Daun02 significantly decreased AIMs compared to baseline score (*p < 0.05; Fig. 2E). AIMs reduction lasted 3 days compared with baseline scores (22%, 21% and 13% respectively; *p < 0.05 for all; Fig. 2E) in keeping with previous demonstration of Daun02-mediated behavioural span^8, 9, 14^. After a return to baseline AIMs scores, a control solution, (vehicle without Daun02), was injected in the dlBST of the same rats. AIMs scores were unaffected in vehicle-treated rats whereas Daun02-inactivation significantly decreased AIMs scores compared to vehicle for 2 days (19% and 18% respectively; ^$^p < 0.05 for all; Fig. 2E). Daun02-inactivation did not affect the rotational behavior, an index of the anti-parkinsonian effect of L-Dopa, compared with both baseline and control-treated rats (Fig. 2F). ß-galactosidase staining confirmed an expression of the FosB-LacZ lentivirus restricted to the dlBST region (Fig. 2G).

## Discussion

More than fifty years after its introduction in clinical therapy, L-Dopa remains the gold standard treatment for PD but rapidly induces fluctuations and LID^1^. Those latter have been associated with both presynaptic and postsynaptic striatal mechanisms although extra-striatal consequences are very likely especially in DA-rich brain regions^1, 17, 18^. In the present study, we unravelled the involvement of an extra-basal ganglia brain region, the dlBST, in LID pathophysiology. First, we demonstrated that the electrical inhibition of FosB/∆FosB dlBST neurons decreased LID severity in the rat model of L-DOPA-induced dyskinesia. Altogether, these results demonstrate, for the first time, a translational validation of the functional involvement of the dlBST in LID, supporting the role of structures outside of the basal ganglia in LID pathophysiology as already suggested by a recent study^9^.

The study is not however without limitations. Experiments were performed in the dyskinetic unilateral 6-OHDA-lesioned rat, which, although it has demonstrated its translational and heuristic values^1^, bears the paradoxical drawback of displaying accurate and reproducible extent and pattern of lesion, at odds with the clinical situation. Thus the modest, though significant, decrease in AIMs after pharmacogenetic inhibition of dlBST in this model calls for caution in hoping to translate this finding in human. Until now, only large reversal of AIM severity has indeed translated into 25–40% reduction of LID in PD patients^1^. In other words, modulation of dlBST activity is unlikely to offer a new target to innovative treatments of LID despite its pathophysiological interest.

The dlBST is a small structure requiring special care when injecting the viral vector. The viral infusions were thus performed under *in vivo* electrophysiological guidance using small volume of virus solution (250 nl) to limit virus particles diffusion to adjacent fiber bundles. That the dlBST is located next to the internal capsula is actually a barrier preventing diffusion, of either the viral vector when injecting it or of daunorubicin itself. We demonstrated that targeting small neuronal ensembles distant from the striatum (e.g. Lateral Habenula) can significantly reduce dyskinesia severity^9^. We found no evidence demonstrating that daunorubicin can be secreted by neurons and then taken up by neighboring passing fibers. If this was occurring, diffusion into fiber bundles following intrastriatal injection of daun02 would result in a pyramidotomy-like effect (e.g. ipsilateral motor deficit). We however previously demonstrated that intrastriatal administration of daun02 alleviates LID and found no evidence for a deleterious effect of daun02 on motor behavior^8^.

The BST receives robust monoaminergic inputs featuring serotonin (5-HT), noradrenaline (NA) and dopamine^19^. The dlBST dopamine inputs originate in the ventral tegmental area (VTA), the periaqueducal gray region and the retrorubral field. They form a fairly diffuse input to the dlBST with dense dopamine terminal fields in the ovBST and the jxBST^20–22^. In addition, the dlBST integrates strong inputs from the central amygdala and insular cortex to modulate motivation and negative emotional states^23^ and adjust information on motor and anxiety-related function via projections to the substantia nigra pars compacta and basolateral amygdala^24^. This anatomical organization has been confirmed in primate and suggests that the dlBST is in a prominent position to modulate dopaminergic circuits involved in initiating movement^25^. Interestingly, previous studies demonstrated that the monoaminergic neurochemistry of the amygdala and cortical areas and IEG-expression pattern are altered in animal models of PD and LID^3, 5^. Consistent with clinical observations, these results suggest that networks involved in affective, anxiety-related and motivational processes, could also impact LID severity either directly or indirectly. Here, we provided behavioral evidences that inactivation of FosB/∆FosB dlBST neurons decreased LID severity. Reduction of anxiety levels has been shown to participate to the reduction of LID severity^26^. Anxiety-related emotional states are controlled by multiple neuronal circuits that share robust and reciprocal connections with the dlBST^27^. In particular, the dlBST tunes anxiety-related function via monosynaptic projections to the basolateral amygdala^24^. In addition to emotional functions processing, the basolateral amygdala also modulates sensorimotor information^28^. Together, these data strongly suggest that changing the activity of the dlBST during expression of LID could have a direct impact on the integration of information related to anxiety and sensorimotor information processing and therefore participate to reduction of AIM severity.

Together with the recent demonstration that neurons in the lateral habenula contribute to the expression of LID^9^, the present study further highlight the cognitive contribution to the expression of abnormal involuntary movements triggered by L-Dopa and identifies some of the underlying networks and mechanisms as shown with the involvement of the dlBST and increased D1R expression.

D1R signaling is enhanced in LID^1^. D1R in LID has been described both as sensitized (increased agonist affinity or increased coupling to Gs/olf per unit time)^1, 29^ and as kept in an active confirmation at membrane for a longer duration^10, 30, 31^. Consequently (or in parallel), LID disturb striatal D1R signalling pathway^32–35^ inducing, among others, alterations in IEG expression, especially for ∆FosB^35^, which impacts LID severity^6–8^. Interestingly, the dlBST shares similar consequences of the chronic L-Dopa treatment with the striatum, with an increase in D1R expression induced exclusively by a dyskinesiogenic chronic L-Dopa treatment, which co-localized with ∆FosB.

In conclusion, the present study describes the involvement of an hitherto unnoticed extra-basal ganglia structure, the dlBST, in LID pathophysiology. Taken together, our results highlight for the first time the functional role of the dlBST in LID.

## Material and Methods

### Study approval

Experiments were performed in accordance with the European Union directive of September 22, 2010 (2010/63/EU) on the protection of animals used for scientific purposes. The Institutional Animal Care and Use Committee of Bordeaux (CE50) approved the present experiments under the license numbers 5012099-A.

### Daun02/ß-galactosidase inactivation method

#### Rat experiments

Adult male Sprague-Dawley rats (Charles River Laboratories, Lyon, France), weighing 175–200 g at the beginning of the experiment, were used (**n** = **12**). They were housed under standard laboratory conditions in a 12-hour light/12-hour dark cycle with free access to food and water. On Day 0, unilateral injection of 6-OHDA (2.5 µl at 3 µg/µl) was unilaterally injected in the right medial forebrain bundle (AP = −3.7 mm; ML = +1.6 mm; DV = −8 mm relative to Bregma), in rats treated 30 minutes before with citalopram (1 mg/kg i.p.) and desipramine hydrochloride (20 mg/kg i.p.) according to previously published procedures^5, 10, 11, 31, 36^. At the same time, all the rats were injected with 250 nl of a lentiviral vector expressing LacZ (coding for ß-galactosidase) under control of a FosB promoter with a final titer of 1.18 × 10^9^ infectious particles/ml, as previously reported^8, 9^, in the dlBST (AP = −0.4 mm; ML = +1.8 mm; DV = −5.6/−7.2 mm). All lentiviral injections were done under *in vivo* electrophysiological guidance whereby the dlBST was located upon specific cortical input stimulation^37^. Stimulation and recording electrodes were respectively inserted into the insular cortex (INS Ctx; AP = −0.2 mm; ML = +5.8 mm; DV = −4.4 mm) and the Ov/JxBST (AP = −0.4 mm; ML = +1.8 mm; DV = −5.6/−7.2 mm), respectively. Bipolar electrical stimulation of the INS Ctx was conducted with a concentric electrode (Phymep, Paris) and a stimulus isolator (500 µs, 0.2–2 mA; Digitimer). Baseline was recorded for 10 min (2 × 100 pulses; 0.5 Hz). Ov/jxBST recordings were done using a glass micropipette (tip diameter, 1–2 µm; 10–15 MΩ) filled with a 2% sky blue pontamine solution in 0.5 M sodium acetate. Extracellular potentials were recorded with an Axoclamp-2B amplifier and filtered (300 Hz/0.5 Kz)^37^. Single neuron spikes were collected online (CED 1401, SPIKE2; Cambridge Electronic Design). During electrical stimulation of the INS Ctx, cumulative peristimulus histograms (PSTHs, 5 ms bin width) of ov/jxBST activity were generated for each recorded neuron. Then, guide cannulas were implanted as previously described^8–10^ (AP = −0.4 mm; ML = +1.8 mm; DV = −5.6/−7.2 mm) and cemented to the skull for subsequent Daun02 injections.

Rats displaying an impaired stepping test^5, 10, 31^ assessed on days 18 to 20 were retained for experiments (n = 10). Lesion extent was also quantified *post*-*mortem* by the loss of tyrosine hydroxylase-immunopositive fibers in the striatum and animals displaying a loss greater than 95% were considered as lesioned. From day 21 onwards, 10 rats received daily an i.p. injection of a combined dose of benserazide (15 mg/kg) and L-Dopa (6 mg/kg) for 10 days. On the 31^th^ day post-6-OHDA and FosB-LacZ lentiviral injections, the baseline abnormal involuntary movements (AIMs) score was assessed. The 4 AIMs categories (limb, axial, orolingual, and locomotive) were scored using a validated rating scale^38, 39^ for 1 minute every 30 minutes for 2 hours (total 4 observations; maximal score for each observation, 16; maximal total score per session, 64) performed by a blinded trained investigator as previously described^5, 10, 31, 36^. The baseline AIMs scores are 38/48 for the cumulated axial, limb and orolingual AIMs scores and 7/16 for the cumulated rotation scores.

On the 32^th^ day, animals (n = 10) received a 6 mg/kg L-dopa injection 1 h before a 500 nl Daun02 injection (4 µg/µL in 5% DMSO, 5% Tween-80 in PBS at 0.5 µl/min)^8, 9^ in the dlBST under light isoflurane anesthesia before being placed in their home cage for 3 days as described^8, 9, 13, 14^. From the 3^rd^ day after Daun02 injection, all rats (n = 10) received a daily 6 mg/kg L-Dopa injection and AIMs were scored^8, 9^. To ensure reversibility of Daun02-induced inactivation, vehicle (5% DMSO, 5% Tween-80 in PBS at 0.5 µl/min) was injected in the same animals 9 days after Daun02 injection and AIMs were evaluated, following the same protocol.

At the end of the Daun02 experiment (day 47, Fig. 1B) and 1 hour after the last L-DOPA injection, i.e. at the peak of behavioural effect, rats were deeply anesthetized with pentobarbital (120 mg/kg, i.p., VWR) and perfused transcardially with 0.9% NaCl followed by ice-cold 4% formaldehyde in PBS. The timeline (Fig. 1B) summarized these 6 weeks of experimental manipulations. Finally, brains were removed, postfixed overnight in the same fixative (4 °C), then cryoprotected for 48 h at 4 °C in 20% PBS-sucrose. Brains were frozen in isopentane at −45 °C and stored at −80 °C until sectioning.

#### In vivo electrophysiological validation of the Daunorubicin-induced electrical inhibition in dlBST neurons

Stereotaxic surgery for *in vivo* electrophysiology, stimulation and recording protocols were done as described above and previously^37^. Local delivery of Daunorubicin (4 µM and 8 µM) or its vehicle (PBS) was done using double barrel pipettes as previously described^37^. Each cell was tested with 100 nL of Daunorubicin or the vehicle. At the end of each recording experiment, the electrode placement was marked with an iontophoretic deposit of sky blue dye (−20 µA, 15 min). To mark electrical stimulation sites, +50 µA was passed through the stimulation electrode for 90 s. Brains were frozen in isopentane and cut with a cryostat (30 µm thick). Sections were mounted with DAPI vectashield medium and observed with epifluorescent and transmission microscopy.

#### Cytochemical detection of β-galactosidase

Coronal sections (50 µm) were collected, washed twice in PBS and incubated overnight at 37 °C in freshly prepared staining buffer [1 mg/mL X-gal (5-bromo-4-chloro-3-indolyl-β-D-galactoside), 5 mM K3Fe[CN]6, 5 mM K4Fe[CN]6, and 2 mM MgCl2 in PBS, pH 6.0] as previously described^8^. Brain sections were washed with PBS, counterstained with neutral red and examined at ×10 and ×40 magnification. The degree of transduction reached in the dlBST was calculated through previous quantifications made with the same lentiviral vector^8^.

### Histological data analysis

Coronal rat brain sections (50 µm) were collected and processed for tyrosine hydroxylase (MAB318, Milipore), ∆FosB (sc-48, Santa-Cruz) and D1R (D2944, Sigma) as previously described^5, 8, 9, 40^.

### Data Analysis

Rat behavioural data were analyzed with Wilcoxon signed-rank t-test^9, 41^. All data are presented as mean ± SEM with a threshold for statistical significance at p < 0.05. For *in vivo* electrophysiological experiments, cumulative PSTHs of ov/jxBST activity were generated during electrical stimulation of the INS Ctx. Excitatory magnitudes (R_mag_ values) were normalized for different levels of baseline impulse activity. In brief, the mean and SD of counts per bin were determined for a baseline period, defined as the 500 msec epoch preceding stimulation. The onset of excitation was defined as the first of 5 bins for which the mean value exceeded mean baseline activity by 2SD, and response offset was determined as the time at which activity had returned to be consistently within 2SD of baseline. R_mag_ values for excitation were calculated according to: Excitation R_mag_ = (counts in excitatory epoch) − (mean counts per baseline bin X number of excitatory bins in excitatory epoch). For a comparison between three groups, values were subjected to a one–way ANOVA followed (if significant) by Bonferroni post hoc tests^42^.
